# Supplementary material for: EnzML: multi-label prediction of enzyme classes using InterPro signatures
Source: BMC Bioinformatics. 2012 Apr 25;13:61. doi: 10.1186/1471-2105-13-61 (PMC3483700; doi:10.1186/1471-2105-13-61)
Supplement: Addtional file 5 — The Java code to format the data files, evaluate and predict. The file enzml_java_code.tar.gz contains the Java code used to format database data to ARFF and XML formats, to execute cross and train-test (jackknife) evaluations and to record evaluation results to database. More information is included in the readme.txt file and the Javadoc files. The code can be used with a MySQL database. To use a different database software, other JDBC drivers might be required. [file 1471-2105-13-61-S5.gz › java_code/enzml2011/doc/uk/ac/ed/inf/enzml/class-use/Initialised.html]

Uses of Class uk.ac.ed.inf.enzml.Initialised


---


|  |  |  |
| --- | --- | --- |
| **Overview** \| **Package** \| **Class** \| **Use** \| **Tree** \| **Deprecated** \| **Index** \| **Help** | |  |
| PREV   NEXT | **FRAMES**    **NO FRAMES**     **All Classes** |  |


---


## **Uses of Class uk.ac.ed.inf.enzml.Initialised**

| Packages that use Initialised | |
| --- | --- |
| **uk.ac.ed.inf.enzml.mulan** |  |
| **uk.ac.ed.inf.enzml.mulan.attributesfilter** |  |
| **uk.ac.ed.inf.enzml.mulan.learn** |  |
| **uk.ac.ed.inf.enzml.mulan.predict** |  |
| **uk.ac.ed.inf.enzml.weka** |  |

Uses of Initialised in uk.ac.ed.inf.enzml.mulan

| Subclasses of Initialised in uk.ac.ed.inf.enzml.mulan | |
| --- | --- |
| `class` | `MulanArff`             Generates an Attribute Relation Format File (ARFF) in Mulan format (multiple class labels per instance). |
| `class` | `MulanArffProperties`             Class |

Uses of Initialised in uk.ac.ed.inf.enzml.mulan.attributesfilter

| Subclasses of Initialised in uk.ac.ed.inf.enzml.mulan.attributesfilter | |
| --- | --- |
| `class` | `AttributesFilter`             This class filters the attributes of a dataset (the test set) to make it compatible with another dataset (the train set). |
| `class` | `AttributesFilteredArff` |

Uses of Initialised in uk.ac.ed.inf.enzml.mulan.learn

| Subclasses of Initialised in uk.ac.ed.inf.enzml.mulan.learn | |
| --- | --- |
| `class` | `MulanArffRecord`             Checks that an ARFF record read from the database contains valid parameters to read an ARFF file. |
| `class` | `MulanCrossExperimenter`             Using Mulan (multi-label machine learning), this class initialises and executes either Train-Test evaluation (if two different arff records are given) (calling `MulanTrainTestEvaluator`) or cross-evaluations (calling `MulanCrossEvaluator`) if only one arff record is given. |
| `class` | `TrainTestExperimenter`             Using Mulan (multi-label machine learning), this class initialises and executes Train-Test evaluation (two arff records are needed, for the training and testing files) (calling `MulanTrainTestEvaluator`). |
| `class` | `TrainTestExperimenterSerialized`             Executes a train/test evaluation using a previously trained model saved on file. |

Uses of Initialised in uk.ac.ed.inf.enzml.mulan.predict

| Subclasses of Initialised in uk.ac.ed.inf.enzml.mulan.predict | |
| --- | --- |
| `class` | `MulanPredict`             First trains on a dataset (training set) and then emits predictions for an unlabelled dataset. |
| `class` | `MulanPredictWithTrainedModel`             Class |

Uses of Initialised in uk.ac.ed.inf.enzml.weka

| Subclasses of Initialised in uk.ac.ed.inf.enzml.weka | |
| --- | --- |
| `class` | `Arff`             Creates an ARFF file: checks file properties, loads data from database, fills and writes to file the ARFF attributes, classes and instances, generates auxiliary files if needed. |
| `class` | `ArffProperties`             Contains the properties for a Weka ARFF file It contains: 1. |

---


|  |  |  |
| --- | --- | --- |
| **Overview** \| **Package** \| **Class** \| **Use** \| **Tree** \| **Deprecated** \| **Index** \| **Help** | |  |
| PREV   NEXT | **FRAMES**    **NO FRAMES**     **All Classes** |  |


---
